# Attentional Orienting in Front and Rear Spaces in a Virtual Reality Discrimination Task

**DOI:** 10.3390/vision6010003

**Published:** 2022-01-06

**Authors:** Rébaï Soret, Pom Charras, Christophe Hurter, Vsevolod Peysakhovich

**Affiliations:** 1 ISAE-SUPAERO, Université de Toulouse, 31400 Toulouse, France; vsevolod.peysakhovich@isae-supaero.fr; 2 Psychology Department, Université Paul-Valéry-Montpellier, 34090 Montpellier, France; pom.charras@univ-montp3.fr; 3 École Nationale de l’Aviation Civile, 31400 Toulouse, France; christophe.hurter@enac.fr

**Keywords:** attentional orienting, Posner paradigm, eye tracking, spatial cognition, rear space, front space, virtual reality, discrimination task

## Abstract

Recent studies on covert attention suggested that the visual processing of information in front of us is different, depending on whether the information is present in front of us or if it is a reflection of information behind us (mirror information). This difference in processing suggests that we have different processes for directing our attention to objects in front of us (front space) or behind us (rear space). In this study, we investigated the effects of attentional orienting in front and rear space consecutive of visual or auditory endogenous cues. Twenty-one participants performed a modified version of the Posner paradigm in virtual reality during a spaceship discrimination task. An eye tracker integrated into the virtual reality headset was used to make sure that the participants did not move their eyes and used their covert attention. The results show that informative cues produced faster response times than non-informative cues but no impact on target identification was observed. In addition, we observed faster response times when the target occurred in front space rather than in rear space. These results are consistent with an orienting cognitive process differentiation in the front and rear spaces. Several explanations are discussed. No effect was found on subjects’ eye movements, suggesting that participants did not use their overt attention to improve task performance.

## 1. Introduction

Attentional processes are largely studied by cognitive researchers in desktop conditions but also in virtual reality [1,2,3,4]. Cueing the target position is an efficient way to study the attentional process since the publication of the influential Posner’s paradigm [5]. In this paradigm, a cue is provided to the participant before the appearance of a target to the left or right of a central fixation point. The cue can be valid (i.e., indicating the correct target occurrence location), invalid (indicating an incorrect target location), or neutral (i.e., indicating all possible positions of target occurrence). Classic results show faster response time (RT) for valid cues than for neutral cues (treatment benefit) and faster response times for neutral cues than invalid cues (treatment cost). The study of this cost/benefit balance makes it possible to determine the impact of a cue on the subjects’ spatial orienting processes. This cost–benefit balance depends on many factors, such as the time interval between the onset of the cue and the target (stimulus onset asynchrony = SOA). For example, for exogenous cues, if the SOA is greater than 300 ms, the cost–benefit balance is generally reversed (RT invalid < RT valid), an effect known as inhibition of return (IOR). The study of this cost/benefit balance makes it possible to determine the impact of a cue on the subjects’ spatial orienting processes. This spatial orienting process can be supported by eye movement (overt attention) or can be performed covertly, without shifting the gaze to the localization predicted by the cue (covert attention). The links between eye movements and covert attention have been the subject of numerous publications, often contradictory (e.g., [6,7]). Additionally, in the studies conducted on covert orienting through the Posner paradigm, participants are instructed to keep their eyes on the central fixation point until the target appears. However, there is rarely a check with an eye tracker to ensure that participants follow the instruction and do not use their eyes (and thus their overt attention) to perform the task. The eye tracker integrated into the head-mounted display (HMD) allows being more rigorous on this point. This eye tracker was also used to record eye response times to measure the possible effect of cues on the subjects’ eye movements.

Mostly presented on a desktop monitor, the stimuli (cues and targets) can also be presented through a virtual reality headset, extending the studied visual field. Recently, it has even been demonstrated that this paradigm can be adapted in a playful virtual reality environment to induce more ecological attentional orienting conditions [8]. The authors used a modified version of Posner’s paradigm in virtual reality to measure the effects of different types of cues (endogenous, exogenous) and modality (visual and auditory) on the deployment of covert orienting during a sandwich-making task and showed similar results to the classical paradigm on standard computer screens. This study paved the way to study the attentional processes in an unrestrained 360° environment when the information can appear all around the user. This means that information can appear outside the visual field of the users and especially behind them, in the rear space. In everyday life, important events can occur anywhere in our environment. For example, sometimes information that requires attention is not directly in front of us. We then have to turn around to process the information, such as when a driver honks his horn behind us and we turn around to see what is happening. To determine whether important information in our environment requires special processing, we can rely on perceptual cues such as the spatial location of sound or the reflection of light on a window. These cues can allow us to direct our attention to events that do not occur directly in front of us, and to prepare the processing of that information so as to respond more effectively. It has long been assumed that the results obtained in the study of attentional orienting in front space could be generalized to rear space [9]. However, recent studies in environments close to the context of these behaviors’ development (ecological condition) have suggested that the mechanisms involved in crossmodal attentional orienting in front space and rear space differ [10]. In 2020, a study in virtual reality suggested that the use of mirrors to reflect information located in the rear space of subjects could allow investigation of the effects of rear space orienting through the Posner paradigm [11]. In this study, participants were asked to destroy, as quickly as possible, space debris that could appear through transparent windows or rearview mirrors. The results show faster response times when targets appear through transparent windows than in the rearview mirror, suggesting differentiated processing when the information perceived in the front space is related or not to specific information behind. This difference in processing between the front and rear spaces still needs to be confirmed, and further investigation is necessary. That is why, in the present study, we wanted to replicate the second experiment carried out by Soret et al. in 2020, with some improvements to study the effect of visual and auditory endogenous cues on the cognitive processes of covert orienting to the rear and front space. We expect a main effect of target location (front/rear) as in the original experiment, the second experiment [11]. Response times when the target occurs in the front should be shorter than response times when the target occurs in the rear.

First, with respect to the unpredictive behavior of the neutral trials (i.e., slower reaction time for neutral than invalid cues) that complicated the cost–benefit analysis of the original experiment [11], we chose not to use an uninformative neutral stimulus for the neutral cue condition (showing all possible positions of target appearance at the same time), but to manipulate the predictivity of the cue (percentage of valid and invalid trials within an experimental block). This means that the cue can be informative (i.e., predicted the target location on every trial, 100% predictive) or uninformative (i.e., predicted the correct target location in half of the trials and the incorrect target location in the other half, 50% predictive). Thus, in predictive blocks, participants were always correctly oriented, whereas, in non-predictive blocks, participants are not oriented, since a non-predictive endogenous cue is unable to orient the participant’s attention (they cannot implement a voluntary orienting strategy because the cue is uninformative). We then have valid (100% predictive) and neutral (50% predictive) conditions. By comparing the response times between these two conditions, we have a measure of the cue’s ability to orient attention without using neutral cues with uncertain behavior [12,13,14]. We expect the main effect of cue predictivity. Subject response times should be faster when the cue is informative (100% predictive) than uninformative (50% predictive).

Second, we introduced a motor response in addition to the ocular response. Indeed, we know that the effects observed through the use of the Posner paradigm (cueing effect, inhibition of return) can vary according to the response required from the participants (saccadic or motor). Thus, in the original experiment, the authors instructed the participant to detect the onset of a target as quickly as possible by performing a saccade. In our study, we asked participants to identify the color of the target and to respond manually using the key associated with the perceived color. We, therefore, replaced the detection task with a discrimination task (choice between two possible responses). We expect similar effects on subjects’ motor response times as those observed by [11], i.e., faster response times when the cue is predictive rather than unpredictive of the target’s location. These effects should also be observed on response accuracy. That is, a higher response accuracy when the cue is predictive rather than non-predictive.

Finally, we wanted to keep the same cue modalities; namely, visual and auditory. Indeed, the first studies that suggested different processing of information in the front and rear space were performed via a crossmodal cueing from hearing to vision [9,10]. Thus, we feel it is important to study both crossmodal and intramodal indenting, which could produce different results [15]. We expect the auditory cue to result in faster responses than the visual cue as already found in the literature [16,17] and the original experiment. In addition, both crossmodal and intramodal guidance should produce a validity effect (predictive RT < non-predictive RT). In other words, the auditory and visual cues should allow participants to direct their attention to the indicated location. In addition, the processing difference related to the target position (front/rear) should be present regardless of the cue modality used (visual/auditory).

## 2. Materials and Methods

### 2.1. Participants

Twenty-one volunteers (8 women, mean age ± SD: 30 ± 8) participated in the study. All had normal or corrected-to-normal vision, no hearing problems, and were native French speakers (auditory cues were presented in French). Following the Declaration of Helsinki, all participants gave their written consent before the experiment. They did not receive any contributions for their participation.

### 2.2. Apparatus

The apparatus was the same as that in the study of Soret et al. [11] and is recalled below. An HTC Vive virtual reality headset with an integrated Tobii eye-tracking system was used. The headset has a Dual AMOLED 3.6${}^{\prime \prime }$ diagonal screen, a resolution of 1080 × 1200 pixels per eye (2160 × 1200 combined), a refresh rate of 90 Hz, and a field of view of 110${}^{\circ }$ (145${}^{\circ }$ diagonally). HTC Vive controllers were used for interaction. The eye-tracking system has a gaze data output frequency (binocular) of 120 Hz with an estimated accuracy of 0.5${}^{\circ }$. The eye tracker has a trackable field of view of 110${}^{\circ }$ (full HTC Vive field of view). The Posner cueing task was developed using the Unity3D game engine supporting C# programming. We also used OpenVR, SteamVR, Tobii eye-tracking plugins, and Tobii Pro SDK.

### 2.3. Stimuli

**Figure 1:**
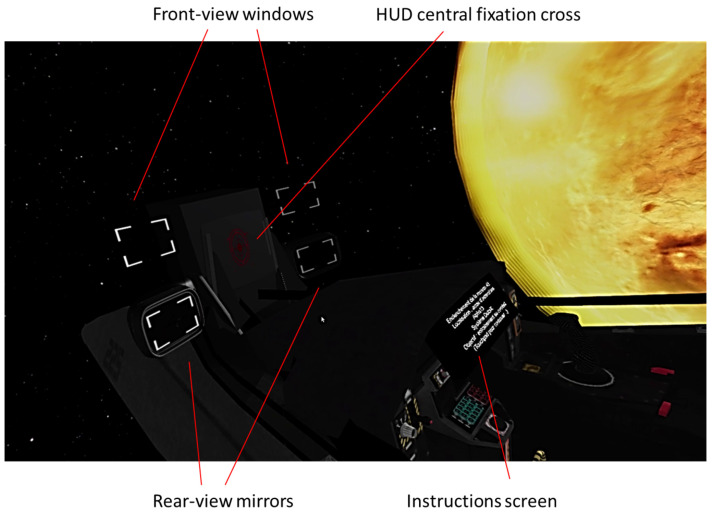
The view of the environment.

Some stimuli were the same as in the study of Soret et al. [11] and are recalled below. Participants were placed in a virtual space environment inside a fighter-like “spacecraft” (see Figure 1). “Aiming assistance tools” were located at the front of the spacecraft to control target occurrence in specific areas of the visual field. These areas were identical in size and shape. This “aiming assistance tool” consisted of a head-up display (HUD) of $4^{\circ }\times 5^{\circ }$ and served as a fixing point. At the four corners of the HUD, there were two horizontally aligned transparent $6^{\circ }\times 13^{\circ }$ viewfinders and two horizontally aligned $6.5^{\circ }\times 13.5^{\circ }$ rearview mirrors. The position of the rearview mirrors and viewfinders was counterbalanced between the subjects to avoid the upper/lower orienting effect on our results. There were rearview mirrors at the top and viewfinders at the bottom, or vice versa, according to subjects.

We used two types of cues: voice instructions corresponding to endogenous audio cues and directional arrows as endogenous visual cues. The voice instructions were the French translation of “Front left”, “Rear left”, “Front right”, and “Rear right” or “Left Front”, “Left Rear”, “Right Front”, and “Left Rear”. Word order was reversed according to the subjects. The arrows were $0.02^{\circ }\times 0.06^{\circ }$. The voice instructions had a duration of 500–600 ms and the directional arrow was displayed for 300 ms. Given that the interstimulus interval (ISI) is 300 ms, the stimulus onset asynchrony (SOA) was 800–900 ms for the voice instruction and 600 ms for the directional arrow. The SOA was not manipulated in the experiment but was chosen to allow sufficient time for participants to interpret it and direct their attention voluntarily (based on [12]).

The target was a spaceship that can be red or blue. If we consider a head-neutral position (looking straight ahead) as the starting point, the frontal space is 90° to the left and 90° to the right; the rear space extends from 90° to 180° to the left and right. The frontal targets were presented at 35° to the left or right, and the rear targets at 145° to the left or the right.

For blocks with 100% predictivity, the cue always indicated the right position of the target. For blocks with 50% predictivity, the cue was valid (indicated the correct target position) for half of the trials and invalid (indicated a wrong position) for the other half. The order of valid and invalid trials was random within the block. For invalid trials, the target could not appear at a directly opposite diagonal position.

The discrimination task consisted of identifying the spaceship color and choosing the proper ammunition. The spaceships to be teleported were blue and those to be destroyed were red. The spaceship lost its color 250 ms after its appearance. Participants had to press the grip key on the right controller to select the destructive ammunition, and the grip key on the left controller to select the teleportation ammunition. The answer keys were counterbalanced according to the subjects.

### 2.4. Procedure

**Figure 2:**
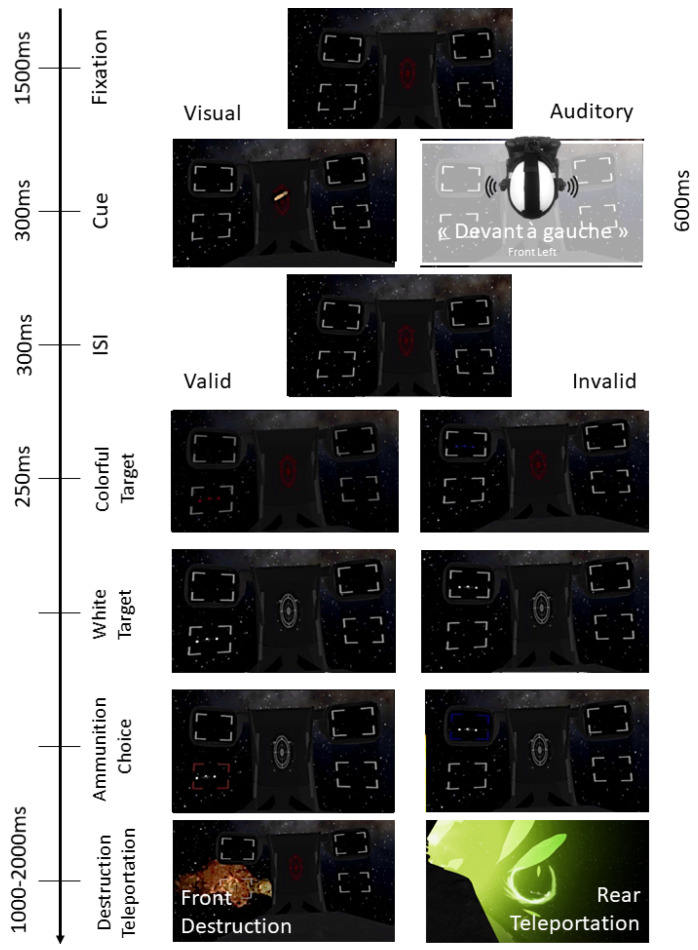
The time course of a trial (from top to bottom).

After completing a consent form, the participants filled out a preliminary questionnaire and read the information sheet. The main instructions were to destroy or teleport the target as quickly and precisely as possible using the provided cue. Before each block, the participants were aware of cue predictivity (100% or 50%) and the assigned answer keys. After fixing the central viewfinder for 1.5 s (fixation point), the participants had to choose the ammunition (destruction/teleportation) by looking at the viewfinder or rearview mirror corresponding to the target’s location and press the correct response key according to the spaceship color. They had to focus and press the trigger button of the controller to destroy or teleport the spaceship for frontal targets (viewfinder) or to turn around, look at the target and press the trigger button of the controller to destroy it for rear targets (rearview mirror). We asked the subject to turn around to “update” their representation of the space around them so that they would not forget that the information perceived in the rearview mirror was located behind them. See Figure 2 for the time course of a trial.

After a 300 ms ISI, the target appeared in one of the 4 possible locations. If participants’ gaze left the fixation point before the target appeared, a new trial was launched. Once the target appeared, participants had to destroy it following the instructions described above. After the destruction of the target, a variable time interval of 1 s to 2 s occurred. The experimental phase consisted of 80 trials: 40 trials per cue type (visual/auditory), divided into 20 non-predictive trials, 10 valid trials (5 front and 5 rear), and 10 invalid trials (5 with target occurrence to the front and 5 to the rear) and 20 predictive trials (10 front and 10 rear). The total duration of the experiment was 30 min, including an experimental session of at least 20 min. This duration was chosen because of virtual reality constraints (eye strain, cybersickness, discomfort) so the participants would go through the experience in the best possible condition without impacting their attention processes. Before the experimental phase, participants performed 16 trials as training (4 trials per cue type and predictivity blocks) to make sure that the instructions were well applied and that they became used to the cues provided.

### 2.5. Data Analysis

#### 2.5.1. Dependent Variable

We recorded two different eye-response times, Gaze-Initiation and Target-Seen, both starting from the target appearance. Gaze-Initiation corresponds to the moment when the participant leaves the fixation point out of sight, and Target-Seen when the participant gazed at the viewfinder/rearview mirror corresponding to the target position (eye response). We also recorded motor response times, Ammunition-Choice, that corresponds to the moment when the participant presses the grip button after a target’s occurrence.

#### 2.5.2. Filter

RTs lower than 0.05 s have been excluded from the analysis because they are beyond the limits of human performance and therefore necessarily represent a measurement error. RTs greater than the meanRT + 2 × std were considered too slow and removed from the analysis (0.887 s for Gaze-Initiation (0.567 + (2 × 0.16)); >1.033 s for Target-Seen (0.639 + (0.197 × 2)); >1.521 s for Ammunition-Choice (0.951 + (2 × 0.285)). One participant was excluded from the analyses for Gaze-Initiation and Target-Seen, due to an excessive number of excluded trials given the criteria defined above.

#### 2.5.3. Trials and Analysis

The analysis includes 72.7 ± 9.3 trials per participant for Gaze-Initiation RTs, 73.0 ± 10.0 trials per participant for Target-Seen RTs, and 71.4 ± 12.3 trials for Ammunition-Choice. We used the JASP software to perform a three-way repeated-measure analysis of variance (RMANOVA) on the average RTs for each dependent variable (Gaze-Initiation, Target-Seen, and Ammunition-Choice) to observe the effect of our manipulated factors: Predictivity (100%/50%), Modality (visual/auditory), and Target Location (Front/Rear). Another RMANOVA was carried out on the responses’ accuracy for Ammunition-Choice with the same experimental factors. Bonferroni correction was used for post hoc comparison.

## 3. Results

### 3.1. Eye Movement

For Gaze-Initiation and Target-Seen, the analysis revealed no significant effects of the manipulated factors after the target occurred. See Table 1 and Table 2 for descriptive statistics.

#### 3.1.1. Gaze-Initiation

The following table shows the mean reaction time for Gaze-Initiation as a function of cues’ modality, predictivity, and location.

#### 3.1.2. Target-Seen

The following table shows the mean reaction time for Target-Seen as a function of cues modality, predictivity, and location.

### 3.2. Accuracy

For Ammunition-Choice, the analysis performed on the accuracy of the responses did not reveal any significant main effect. However, there was an interaction between the modality (visual/auditory) and target location (front/back) factors (F(1,20)=1.817, p<0.001, $\eta^{2}=0.035$). Post hoc comparisons revealed that the response accuracy is significantly higher for auditory cues than for visual cues when the cue appears in front (p=0.006,MD=0.045). Moreover, there is significantly greater response accuracy when the target appears in front than in the rear for the visual cue (p=0.036,MD=0.012).

### 3.3. Response Time

**Figure 3:**
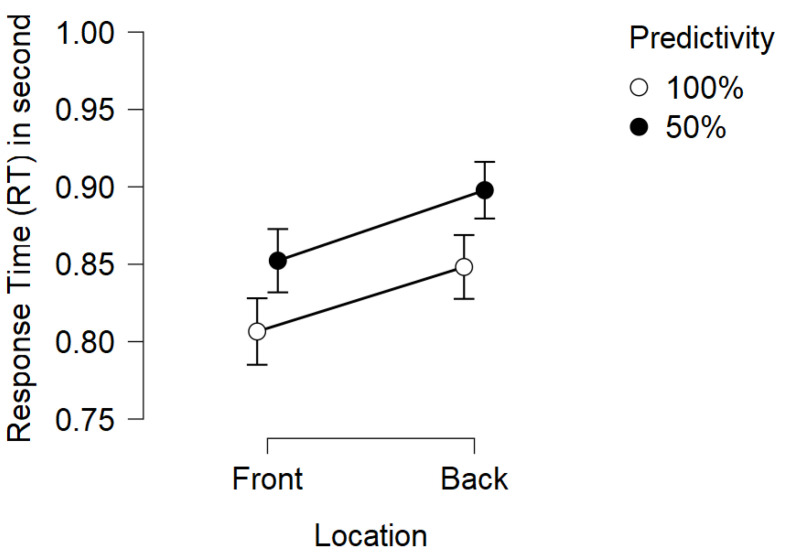
Mean RTs for Ammunition-Choice in response to auditory cues according to cue predictivity and cue-target location. Bars represent standard error.

**Figure 4:**
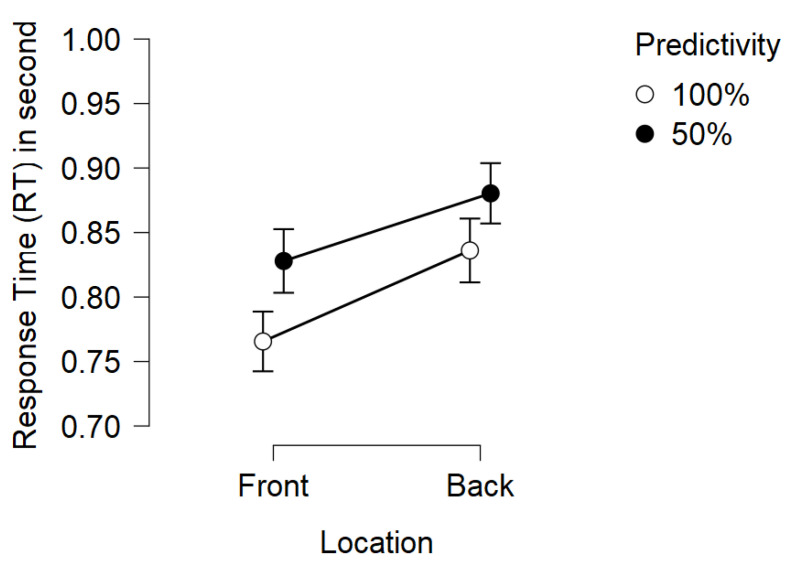
Mean RTs for Ammunition-Choice in response to visual cue according to predictivity and cue-target location. Bars represent standard error.

Concerning response times, the analysis revealed a significant main effect of predictivity on subject response time, as expected ($F\left(1,20\right)=8.390,p=0.009,\eta^{2}=0.063$). Participants obtain significantly shorter average response times in blocks where the cue is always valid compared to blocks where the cue is invalid half the time (MD = 50 ms). Additionally, there was a significant main effect of target location on subject response time ($F\left(1,20\right)=7.392,p=0.013,\eta^{2}=0.068$). The average response time of the participants is significantly faster when the target appears in front of them rather than in the rear (MD = 53 ms). No other significant effects were observed (see Table 3). For an illustration of the results see Figure 3 and Figure 4. For descriptive statistics see Table 4.

## 4. Discussion

The objective of this study was to investigate the differences in attentional orienting in front and rear space. For this, we used a modified version of the Posner paradigm in virtual reality during a spaceship discrimination task. We used two types of cues to orient subjects’ attention: a directional arrow and a vocal instruction. We also manipulated the cue predictivity which could be predictive (100% valid cue) or not (50% valid cue, 50% invalid cue). We carried out three measurements: the initiation of eye movement towards the target after its onset (i.e., the moment when the person moves their eyes from the central fixation point), the interval between the target’s onset, the moment when the target is looked at openly, and finally the moment when the person selects the ammunition to be used after the target’s onset.

### 4.1. Eye Movements

Regarding eye movement initiation towards the target and the moment when the person looks at the target, we do not observe any significant effect of the cue predictivity, contrary to [11]. There are several possible explanations for this lack of effect on the subjects’ ocular responses. The first is that manipulating predictivity instead of validity does not reveal any effect on eye movements. Indeed, we did not compare trials in which the cue directs participants’ attention efficiently or not (valid trial vs. invalid trial) but blocks in which the cue did or did not allow spatial orienting. Thus, the effects usually observed on ocular measures when comparing valid and invalid trials may be more difficult to observe via the manipulation of predictivity.

Moreover, in terms of the premotor theory of attention, which assumes a pre-activation of the motor steps necessary for the action to be performed, since attention is derived from the planning of different motor activities, its properties would depend on the type of motor activity that is planned [18]. For example, a differential effect of attention on ocular and motor responses has been shown by [19]. Moreover, the inhibition of return effect (RT invalid < RT valid), observed by using the Posner paradigm, varied if participants were instructed to respond by pressing a response key (motor response), with saccadic eye movements (saccadic response), or both (motor and saccadic response) [12,20]. In our study, we asked participants to look at the target and press a key to lock it. Thus, we asked them for an ocular and motor response, whereas, in the original experiment, only an ocular response was asked of the participant. The addition of a motor response could be an explanation for the differences observed in the ocular measures between the experiment of [11] and this one.

Moreover, in our study, the participants had to look at the target to lock it. However, they could perform their motor response before or after looking at the target. Thus, they were able to privilege the motor response over the ocular one. The participants may have focused on the primary task, discriminating the color of the target. Thus, participants were able to focus only on the motor response and response accuracy and not on their gaze, so saccadic response was not affected in this task. The nature of the task (discrimination vs. detection) could also be an explanation. Indeed, in the original study, the authors used a detection task, whereas we used a discrimination task in this study. Thus, by changing the task, the motor aspect necessary for a good response may have more impact in our experiment than when using a simple detection task.

In addition, we wished to study covert endogenous attention. Therefore, participants were instructed not to move their eyes until the target appeared. This instruction may have discouraged participants from making a saccadic preparation towards the target to prevent missing the trial, which was otherwise restarted automatically thanks to the monitoring of an eye tracker. Moreover, it would seem that a saccadic preparation is not necessary for the deployment of endogenous attention [21]. However, in our study, we use only endogenous cues. In the study conducted by Soret et al. in 2020, they also used endogenous cues. The fact that they asked only for an ocular response from the participant may have generated a saccadic preparation that otherwise would not occur.

Finally, this lack of effect on the participants’ eye movements upon target onset, added to the monitoring via the eye tracker embedded in the HMD before its onset, shows that the participants did not derive any information processing benefit via eye movement (overt attention), but used their covert orienting ability to prepare for information processing towards the cued region of space.

### 4.2. Motor Response Times and Accuracy

#### 4.2.1. Predictivity Effect

Regarding motor response times, we observe as expected a main effect of the cue predictivity. Participants responded faster when the cue was informative (100% predictive) than when it was not (50% predictive). This means that participants process information faster when they can effectively orient their attention. However, no effect of the cue’s predictivity was observed on response accuracy (ACC). Apparently, in this experiment, although effective attentional orienting allowed participants to respond more quickly to targeted information, it did not allow them to improve target identification. Nevertheless, since the rate of correct responses was particularly high (average ACC = 94%), the discrimination task might not have been difficult enough to highlight a difference (cueing effect). Future experiments should test this hypothesis by increasing task difficulty.

However, we note that the auditory cue seems to provide better accuracy than the visual cue when the target appears through the transparent windows and that no modality difference is observed when the target appears in the mirrors. It also appears that the visual cue provides better accuracy when the target appears through the transparent windows than through the mirrors. These results are unexpected and surprising. It seems premature to make any interpretation or conclusions about these findings. Further investigation is required.

Finally, we note the absence of a modality effect on the response times of the subjects, contrary to [11]. Generally, we observe faster response times for auditory cues than for visual cues [17,22], and this effect is often explained by the fact that auditory cues are more alerting than visual cues (e.g., [23]). Nevertheless, sometimes this difference between visual and auditory cues is not apparent (see, for example, [24]). Given that the previous experiment [11] had revealed a modality effect, the source of this difference could be sought for in the task performed (detection/discrimination). The beneficial alerting effect of auditory cues may be more impactful in a detection task than in a discrimination task. Another reason for the lack of modality effect could be the sample size and the smaller number of trials in our experiment than in the original experiment (20 participants vs. 33 participants; 80 trials vs. 120 trials).

#### 4.2.2. Target Location Effect

The results show the main effect of the target location. Participants reacted more quickly when the target appeared through the clear windows (front) rather than in the rearview mirrors (rear). Similar to the results of [11], there appears to be a difference in processing when information is associated or not with another information outside the frontal space of the subjects. One possible explanation is that there is a difference in the attentional orienting process in the rear space and the front space as suggested in the studies on crossmodal orienting [9]. Indeed, in our study, we find an additive effect of cue predictivity and target location. This additive effect, which is manifested by two main effects without interaction, suggests that attentional engagement (predictivity) and target location in space (front, rear), act on different processing steps, according to Stenberg’s [25,26] additive factor method. Thus, orienting our attention in front and rear space would not act on the same processing steps as cue predictivity. To validate this hypothesis, further studies are needed, notably by recording neurophysiological measures such as electroencephalography (EEG) or functional magnetic resonance imaging (fMRI).

This difference could lie in the spatial representation needed when we direct our attention towards a selected region of space. Indeed, we know that we use two types of spatial frames to encode the surrounding space: an egocentric spatial frame, where the position of objects is coded in relation to the subject’s position, and an allocentric spatial frame, where the position of objects is coded independently of the subject’s current position and centered on the objects present in the environment [27,28]. It is possible that when information is perceived through a mirror (or a rearview mirror), we need to make a change in the spatial frame to determine the actual location of the object to be processed. Indeed, to direct our attention, we need to determine the region of space to prioritize by calling a representation of the space associated with this region. Each frame of reference creates its own representation. When we perceive a piece of information directly, the call to an egocentric spatial representation is sufficient. Nevertheless, when we perceive information through a mirror, we might have to pass from an egocentric representation (where the mirror is in relation to us) to an allocentric representation (where the mirror is in relation to the information that we have to process) to determine the region of space that requires a privileged treatment. Thus, the response times are impacted and are slower than when the information is perceived directly in front of us. It would be interesting for future studies to investigate this possibility.

Another explanation for this difference could be due to a processing benefit for the front space due to long-term learning. Indeed, we can assume that, in everyday life, we tend to direct our attention in front of us, as visual information is first perceived in front of us before being integrated into a 360° representation of the surrounding space. Thus, the experience of a lifetime in front space compared to the relative rarity of orienting behaviors in rear space could be at the origin of this difference. However, in this case, it is unclear why no interaction between target location and validity was observed. If the difference in treatment between the front and rear space was due to a treatment bias for the front space, the effect of predictivity would be expected to interact with the target location, with a stronger effect in the front space than in the rear space, or even the absence of a location effect when the person is unable to orient effectively (neutral trial). This was observed on saccadic response times in [11] with no effect of localization for the neutral and invalid trials. It is possible that manipulating cue predictivity did not reveal an interaction that might have arisen, for example, by using valid and invalid trials with 80% validity throughout the experiment rather than two separate experimental blocks of 100% and 50% predictivity. Future studies may address this issue. Finally, to study this hypothesis of a frontward bias, it would be interesting to explore the effect of video game or a car driving expertise on the observed differences in front and rear space. For example, we can expect that people with experience in playing video games will have faster response times for frontal information than non-gamers, while car drivers who are used to processing information via rearview mirrors should perform better in the rear than non-drivers.

To better understand the origin of the observed differences between the front and rear spaces, the number of trials and subjects could be increased to improve statistical power and test the effects of practice on the observed results. Indeed, in our study, participants were relatively naive and had little experience with rapid information processing in virtual reality and through mirrors. Longer sessions with longitudinal tests could tell us more about the determinants of differences in attentional orienting to front and rear space and its evolution over time.

## 5. Conclusions

In summary, the voice instruction (an endogenous auditory cue) and directional arrow (endogenous visual cue) allowed the participant to determine the color of the spaceship (discrimination task) faster when it was informative about the spaceship’s onset location than when it was not (cue predictivity). However, cue predictivity did not appear to affect the accuracy of responses. Moreover, it appears that targets perceived in front of the subjects but associated with information located behind them (reflective information using a rearview mirror) impacted the processing speed of these targets compared to purely frontal information.

This question of the difference in processing in front and rear space is essential to understand the attentional orienting processes involved in everyday life. Indeed, studies conducted in the laboratory are far from the real conditions of behavioral elaboration, and we still ignore the complex dynamics of cognitive processes in real life. The use of virtual reality to study attentional orienting mechanisms allows us to explore orienting processes in an environment that gains ecological validity and expands the subject’s space. Because of this additional spatial dimension, it is important to consider the subjects’ entire field of view (FOR, 360° in virtual reality) and not just a small portion of the field of view (FOV, typically 60° in the laboratory). The FOV is the size of the visual field in degrees that can be seen instantaneously [29], whereas FOR is the total area that can be seen by turning the head and moving the body [30]. The FOR has been neglected in studies of human perception. Yet, it is important in the real world, as we constantly move our heads and bodies to explore our environment and develop our behaviors [31]. Thus, we can hope to understand how we direct our attention daily when information may arise in the entirety of our perceptual environment and not just in a small part of the frontal visual field. However, if attentional orientation in front and rear space seems to require different processing steps, many questions remain and further investigations are needed.

The exploration of these differences and the results obtained by using a modified version of Posner’s paradigm with reflexive information could lead to the development of guidance systems using reflex information to direct individuals’ attention towards important events requiring processing beyond the directly perceived frontal space. For example, in situations with high attentional demand and which sometimes require extremely fast reaction times to guarantee the safety of people such as driving, flying, or medical emergencies.

## Figures and Tables

**Table 1:** Mean RT (in seconds) as a function of experimental factors for Gaze-Initiation.

Modality	Predictivity	Location	Mean	SD	N
Auditory	50%	Front	0.501	0.109	20
		Rear	0.515	0.109	20
	100%	Front	0.497	0.111	20
		Rear	0.526	0.136	20
Visual	50%	Front	0.476	0.128	20
		Rear	0.514	0.089	20
	100%	Front	0.475	0.125	20
		Rear	0.507	0.085	20

**Table 2:** Mean RT (in seconds) as a function of experimental factors for Target-Seen.

Modality	Predictivity	Location	Mean	SD	N
Auditory	50%	Front	0.518	0.125	20
		Rear	0.520	0.116	20
	100%	Front	0.509	0.129	20
		Rear	0.544	0.139	20
Visual	50%	Front	0.480	0.137	20
		Rear	0.521	0.095	20
	100%	Front	0.486	0.138	20
		Rear	0.511	0.089	20

**Table 3:** Within subjects effects for Ammunition-Choice according to Predictivity (100, 50), Modality (visual, auditory), and Location (front, rear).

Cases	Sum of Squares	df	Mean Square	F	*p*	η ${}^{2}$
Modality	0.024	1	0.024	1.337	0.261	0.014
Residuals	0.354	20	0.018			
Validity	0.107	1	0.107	8.390	0.009	0.063
Residuals	0.255	20	0.013			
Location	0.116	1	0.116	7.392	0.013	0.068
Residuals	0.314	20	0.016			
Validity * Modality	<0.001	1	<0.001	0.089	0.769	<0.001
Residuals	0.314	20	0.016			
Modality * Location	0.003	1	0.003	0.211	0.651	0.002
Residuals	0.319	20	0.016			
Validity * Location	<0.001	1	<0.001	0.133	0.719	<0.001
Residuals	0.080	20	0.004			
Validity * Modality * Location	0.001	1	0.001	0.490	0.492	<0.001
Residuals	0.080	20	0.004			

**Table 4:** Mean RT (in seconds) as a function of experimental factors for Ammunition-Choice.

Modality	Predictivity	Location	Mean	SD	N
Arrow	50%	Rear	0.880	0.214	21
		Front	0.828	0.172	21
	100%	Back	0.836	0.181	21
		Front	0.766	0.190	21
Voice	50%	Rear	0.898	0.234	21
		Front	0.852	0.240	21
	100%	Rear	0.848	0.211	21
		Front	0.807	0.215	21

## Data Availability

The data presented in this study are available on request from the corresponding author.
